# Increasing prevalence of mental disorders in smokers seeking treatment of tobacco dependence: a retrospective observational study

**DOI:** 10.1186/s12888-023-05115-x

**Published:** 2023-08-24

**Authors:** Kamila Zvolska, Ales Tichopad, Lenka Stepankova, Alexandra Pankova, Zuzana Adamcekova, Gleb Donin, Jakub Rafl, Eva Kralikova

**Affiliations:** 1 Centre for Treatment of Tobacco Dependence, the 3rdMedical Department – Department of Endocrinology and Metabolism, First Faculty of Medicine, Charles University, General University Hospital, Prague, Czech Republic; 2 Department of Biomedical Technology, Faculty of Biomedical Engineering, Czech Technical University, Prague, Czech Republic; 3 Institute of Hygiene and Epidemiology, First Faculty of Medicine, Charles University, General University Hospital, Prague, Czech Republic

**Keywords:** Tobacco dependence, Treatment, Smoking cessation, Psychiatric disorders, Prevalence, Comorbidity

## Abstract

**Background:**

There has been a noticeable relative increase in psychiatric comorbidities among smokers as opposed to the general population. This is likely due to comparatively slower decrease in smoking prevalence among individuals with mental health conditions. This study aims to assess the prevalence trend of past or current mental health disorders in individuals seeking specialized smoking cessation assistance.

**Methods:**

We conducted a retrospective single-centre observational study to assess the presence of mental disorders such as anxiety, depression, bipolar affective disorder, or schizophrenia in personal history of 6,546 smokers who sought treatment at the Centre for Treatment of Tobacco Dependence in Prague, Czech Republic between 2006 and 2019. The study examined the impact of gender, age, and the effect of successive years on the prevalence of the mental disorders using Poisson distribution regression.

**Results:**

In the studied cohort, 1,743 patients (26.6%) reported having one or more mental disorders. Compared to patients without a psychiatric disorder, they exhibited similar levels of carbon monoxide in expired air (mean 17 ppm, SD 11 ppm) and scored one point higher on the Fagerström Test of Cigarette Dependence. Among smokers with a mental disorder, women were more prevalent (62%) than men (38%). The prevalence of mental disorders increased on average by 4% every year, rising from 23% in 2006 to 35% in 2019.

**Conclusions:**

Consistent with the observation that the prevalence of smoking among people with any mental disorder is higher and declining at a slower rate than in the general population, there is a steadily increasing percentage of these patients seeking specialized treatment over time. Professionals who offer tobacco dependence treatment should be aware of the upward trend in psychiatric disorders among smokers, as more intensive treatment may be needed. Similarly, psychiatric care should pay attention to smoking of their patients.

## Background

People with mental disorder smoke more often and more intensively compared to general population [1]. In the USA, it was reported that about 40% of cigarettes were sold to people with a mental disorder in 1990, while the smoking prevalence was 28% [2–4]. Similarly, more than half of all cigarettes sold in Australia, New Zealand, and the US in 2016 were sold to people with a mental disorder [4, 5]. In 2019, the prevalence of smokers reporting past-month cigarette use with any past-year mental disorder was 1.8 times higher than those without a mental disorder in the USA. There was 1.8 times higher prevalence of smokers reporting past-month cigarette with any past-year mental disorder than those without mental disorder reported in the USA in 2019 [6, 7]. Presumably, more than 60% of people with schizophrenia and as many as 33–70% of people with bipolar disorder smoke tobacco [8, 9].

Cigarette smoking increases the risk of mortality from cardiovascular disease in people with schizophrenia [10]. Overall, tobacco-related diseases are the most frequent cause of death of persons with mental disorder, accounting for approximately 50% of deaths among people with schizophrenia, bipolar disorder, and depression [11, 12].

Smoking cessation is generally associated with improved mental health [13]. There are indications that more difficult access to a treatment of tobacco dependence may contribute to the reported slower decline in smoking prevalence over time in people with mental disorders [1, 14]. The EAGLES study provides evidence that standard pharmacological and behavioural treatments, with modest adaptations, can be used safely and effectively in persons with a mental disorder [15]. It is therefore important that effective smoking cessation strategies are used to help people with severe mental disorders to stop smoking [1].

In the general Czech population, almost 30% have some mental disorder [16]﻿. In the Czech Act on Protection of Health against the Harmful Effects of Addictive Substances prohibits smoking in public indoor spaces; however there is an exception that allows for a structurally separated area reserved for smoking in a closed psychiatric ward or other addiction treatment facility [17].

To date, little is known about the proportion of patients with a mental disorder among smokers seeking intensive treatment for tobacco dependence. This study aimed to address the research question regarding the temporal trend in prevalence of patients with a mental disorder among smokers seeking specialized tobacco dependence treatment.

## Methods

The single-centre retrospective observational study was conducted at the Centre for Treatment of Tobacco Dependence of the 3^rd^ Medical Department at the General University Hospital in Prague, Czech Republic (Centre). The publication of results of our standard treatment of tobacco dependence was approved by the Ethics Committee of General University Hospital in Prague No. 30/13, 49/21, and 1254/22 IS, D. At baseline visit, all patients signed informed consent and agreed to the evaluation of their personal data in anonymised form for research purposes.

### The study site

The Prague-based Centre provides an intensive specialized tobacco dependence treatment to, roughly, 400–500 patients each year and operates full time for smokers since 2005. Patients could have been referred to the treatment Centre by their physician or self-refer. The treatment is based on evidence-based guidelines and has typically been tailored to the individual patient’s needs [18–20]. The treatment is provided by a nurse and a physician both with completed professional medical training, and it consists of face-to-face counselling – psychobehavioural intervention and mostly recommended pharmacotherapy including nicotine replacement therapy, varenicline, cytisine, and/or bupropion. In frame of the personal history collection, patients were asked about mental disorders, and positive answers were sorted in the following categories: anxiety, depression, bipolar affective disorder, or schizophrenia. The diagnoses were not verified by psychiatrist. The initial psychobehavioural intervention lasted about 2 h and subsequent visits about 30 min, with 12-months follow-up as described elsewhere [21].

### The treatment cohort and the data collection

The analysed dataset included 7,498 patients treated as outpatients from 2005 to 2021 during the 12-month follow-up period, with an average number of visits being 4.3 (SD 2.7). The participants had an average age of 44.0 years (SD 14.0 years), with 52% male and 48% female. All individuals were aged 18 or older, heavily dependent on smoking, with an average of 21.7 cigarettes/day (SD 11.3 cigarettes/day) and average score of 5.5 points (SD 2.4 points) in the Fagerström Test of Cigarette Dependence (FTCD) [22, 23].

For the sake of consistency, records from the years 2005, 2020 and 2021 were excluded. Additionally, individuals with missing key information during the overall period were excluded. Thus, for this analysis, we included only those with the date of the first visit falling before the start of COVID-19 epidemic (2006–2019), resulting in a sample size of *N* = 6,546.

The baseline visit lasted approximately 1 h and involved a basic medical examination along with data collection on demographics, smoking, smoking dependence characteristics, personal smoking and medical history, and self-reported psychiatric problems, current or past, such as anxiety, depression, bipolar affective disorder, schizophrenia, including the year of onset and psychiatric pharmacological treatment. The Beck Depression Inventory (BDI-II) was also administered during the baseline visit [24, 25].

### The statistical analysis

The data was described by means of the descriptive statistics in the software R v. 4.1.1 for Windows. Age groups were created as below 26, 26–35, 36–45, 46–55, 56–65, and above 65. Trend in the prevalence of reported mental disorders was analysed using the multivariate Poisson regression analyses of the R v. 4.2.1 for Windows package.

The year of the initial visit of the smoker in the Centre was taken as either metric covariate or a factor to better illustrate the year-to-year trend. In the either case, point and interval estimates of rate ratios associated with potential covariates were calculated and plotted as based on the multivariate generalized linear model with log-link function and Poisson distribution [26]. To obtain counts (*N*) by each factor combination, the records were grouped by the aforementioned age groups, gender, and the year of the first visit.

The general multivariate Poisson regression model with age as a covariate and gender as additional analysed factor was constructed as follows:

1 $$\mathrm{log}{N}_{mental\_disorder}={\beta }_{0}+{\beta }_{1}Age+{\beta }_{2}{Gender}_{i}+{\beta }_{3}Year+\mathrm{log}{N}_{total}$$

where the *i* denotes the gender and the year is studied as a continuous covariate. Alternatively, to understand the effect of individual years, the model was also fitted as:

2 $$\mathrm{log}{N}_{mental\_disorder}={\beta }_{0}+{\beta }_{1}Age+{\beta }_{2}{Gender}_{i}+{\beta }_{3}{Year}_{j}+\mathrm{log}{N}_{total}$$

where the *i* denotes the gender and the *j* the respective year studied as a factor. Both regression models estimate the prevalence rate ratio (PRR), the associated 95% confidence interval (CI), and the *p*-value as presented in Table 2.

## Results

### Cohort description

The full dataset of 7,498 individuals, collected from January 2005 through 31 December 2021 was cleaned for consistency and completeness, resulting in an analysis set of patients who attended an initial visit during the period from 2006 to 2019. Among the total of 6,546 patients, 1,743 (26.6%) reported one or more of the above-mentioned mental disorders—19,6% of men (668/3,400) and 34.2% of women (1,075/3,146). The details of the study cohort are presented in the Table 1. The total number of patients attending the initial visit was 500 or more each year during 2006–2009 with a downward trend in the following years except for 2017. The proportion of women attending their initial visit oscillated between 43 and 52% each year. Among patients with a mental disorder, women were more prevalent (62%) than men (38%). The average age of smokers at the baseline visit was 44 years (SD 14), both for smokers with and without a mental disorder. Similarly, there was no difference in the average number of cigarettes smoked per day – both groups reported an average of 22 cigarettes per day (SD 11). Additionally, the baseline CO levels of 17 ppm (SD 12) did not differ between both groups. However, the median FTCD score was significantly higher in those with a reported mental disorder (6 vs. 5 with IQR 4 vs. 3, respectively).

**Table 1:** Cohort of smokers presenting at the Centre from 2006 through 2019

	**Total** *N* = 6,546^a^	**Psychiatric diagnosis**		*p*-value^*^
		**No** *N* = 4,803^a^	**Yes** *N* = 1,743^a^	
**Gender**				< 0.001
**Male**	3,400 (52%)	2,732 (57%)	668 (38%)	
**Female**	3,146 (48%)	2,071 (43%)	1,075 (62%)	
**Age (years)**	44 (14)	44 (14)	44 (13)	0.13
**Number of cigarettes per day**	22 (11)	22 (11)	22 (13)	0.055
**Baseline CO (ppm**^b^**)**	17 (12)	17 (12)	17 (11)	0.3
**FTCD score**	6.00 [4.00–7.00]	5.00 [4.00–7.00]	6.00 [4.00–8.00]	< 0.001

^a^n (%); Mean (SD); Median [IQR]

^b^ppm, parts per million

^*^Pearson's Chi-squared test; Wilcoxon rank sum test

### Data grouping

The 6,546 records grouped by age group, gender, and the year of first visit resulted in a contingency table of 168 groups with, on average, 39 (ranging from 2 to 119) records per group. The total number of patients (${N}_{total}$), the number of patients with mental disorder (${N}_{mental\_disorder}$), and the proportion of mentally ill patients were then calculated for each group to populate the regression model.

### Regression analysis

**Fig. 1:**
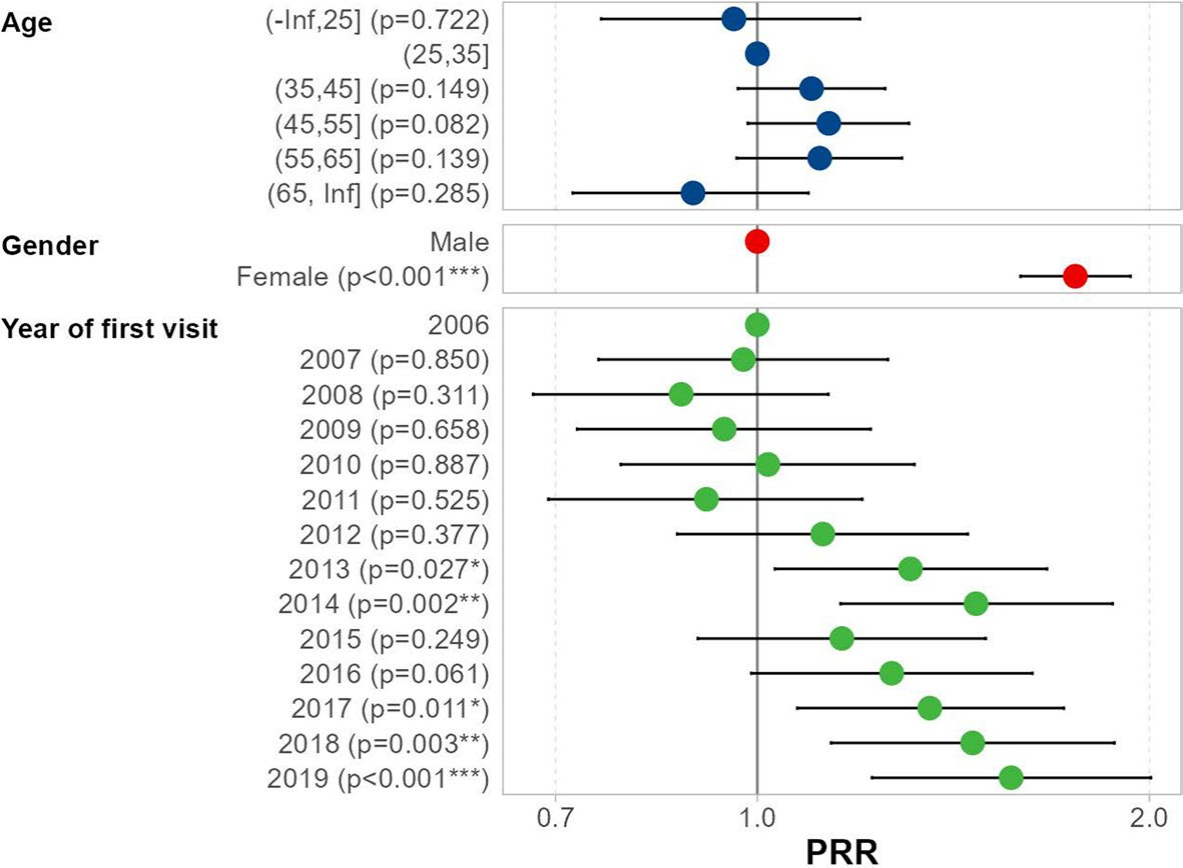
Prevalence Rate Ratio (PRR) with associated 95% confidence interval based on the multivariable Poisson regression model with the year of the first visit as a factor to accentuate the trend of the increasing prevalence of the persons with a mental disorder among smokers

**Fig. 2:**
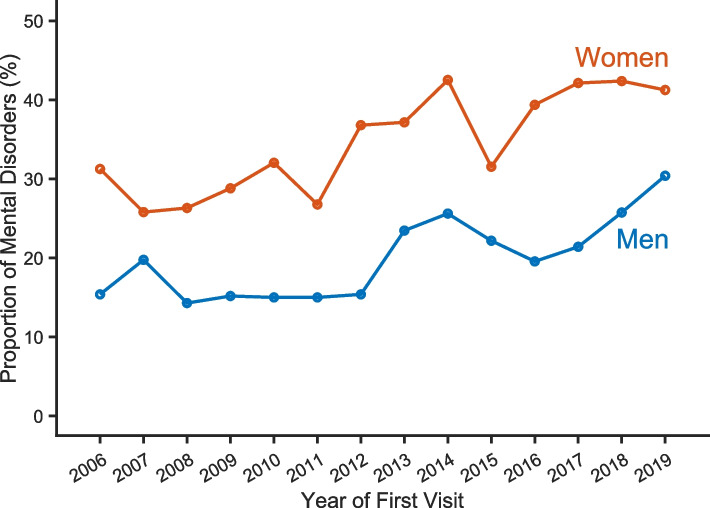
The proportion of persons with a mental disorder among women and men patients. Total number of patients 2006–2019 = 6,546; 529 (2006), 500 (2007), 541 (2008), 500 (2009), 471 (2010), 413 (2011), 446 (2012), 496 (2013), 453 (2014), 443 (2015), 446 (2016), 548 (2017), 379 (2018), and 381 (2019)

As estimated by the model with the year as a continuous covariate (Eq. 1, Table 2), there is a rather consistent 4% annual increase (CI 3% to 5%) in prevalence of individuals with a mental disorder among those seeking help in the Centre. The plot based on the model with year as a factor (Eq. 2, Table 3, and Fig. 1) shows that after an initially oscillating trend between year 2006 and 2011, the prevalence rumps up, and five of the eight years following 2011 show significantly higher prevalence of individuals with mental disorder. In 2019, the prevalence of smokers with a mental disorder in the Centre was 52% higher than in 2006 (increased from 23 to 35%). The proportion of female individuals with a mental disorder was significantly higher than the proportion of male individuals with a mental disorder (*p* < 0.001), as illustrated in Fig. 2.

**Table 2:** The Poisson regression model with the year of the first visit as a continuous covariate

Parameter	PRR estimate	95% CI	*p*-value
Age			
(-Inf,25]	0.95	0.75, 1.19	0.7
(25,35]	1.00	—	
(35,45]	1.11	0.97, 1.26	0.12
(45,55]	1.14	0.99, 1.31	0.069
(55,65]	1.13	0.98, 1.31	0.093
(65, Inf]	0.91	0.73, 1.11	0.3
Gender			
Female	1.75	1.59, 1.93	< 0.001
Male	1.00	—	
Year of first visit	1.04	1.03, 1.05	< 0.001

**Table 3:** The Poisson regression model with the year of the first visit as a factor

Parameter	PRR	95% CI	*p*-value
Age			
(-Inf,25]	0.96	0.76, 1.20	0.7
(25,35]	1.00	—	
(35,45]	1.10	0.97, 1.25	0.15
(45,55]	1.13	0.98, 1.31	0.082
(55,65]	1.12	0.96, 1.29	0.14
(65, Inf]	0.89	0.72, 1.09	0.3
Gender			
Male	1.00	—	
Female	1.75	1.59, 1.93	< 0.001
Year of first visit			
2006	1.00	—	
2007	0.98	0.76, 1.26	0.9
2008	0.87	0.67, 1.13	0.3
2009	0.94	0.73, 1.22	0.7
2010	1.02	0.79, 1.32	0.9
2011	0.91	0.69, 1.20	0.5
2012	1.12	0.87, 1.45	0.4
2013	1.31	1.03, 1.67	0.027
2014	1.47	1.16, 1.87	0.002
2015	1.16	0.90, 1.50	0.2
2016	1.27	0.99, 1.63	0.061
2017	1.36	1.07, 1.72	0.011
2018	1.46	1.14, 1.88	0.003
2019	1.57	1.22, 2.01	< 0.001

There is no obvious consistent trend between age and the prevalence of those with mental disorder. However, it seems that the prevalence of mental disorders is, roughly, high among those aged 36 to 65, followed by a drop in those 66 of age and older. This observation is however only speculative and is not supported by statistical inference.

## Discussion

We found that the prevalence of smokers with a mental disorder who sought treatment was increasing year by year since 2011. Although there is predominance of males who smoke in the general population and among patients with mental disorders [27], our analysis showed a preponderance of females with mental disorders. Patients with and without a mental disorder did not differ in average age, CO level and number of cigarettes per day. The only significant difference was observed in the median FTCD score, which was higher in those who reported a mental disorder. This is in line with the findings of other studies, where the level of tobacco dependence tends to be more intense in people with a psychiatric disorder [28]. Psychiatric disorders such as anxiety, bipolar disorder, insomnia, major depressive disorder, posttraumatic stress disorder, suicide attempts, and schizophrenia are common among smokers. Furthermore, recent studies have indicated that these disorders, aside from psychosocial aspects, may have genetic relationships [29]. Treatment outcomes in these patients tend to be worse compared to general population, especially for depression, where its presence at the start of treatment may predict reduced smoking abstinence after one year. However, a considerable improvement in depression was found in patients who successfully quit smoking and maintained one year of abstinence [30, 31].

It was reported that smoking prevalence in the Czech population aged 15 and older and including both daily and occasional smokers decreased from 31% in 2004 [32] to 24% in 2021 [33]. This trend can be understood as the result of a selective two-speed process where the general population increasingly abstains from smoking, but this occurs more slowly or not at all in those with a mental disorder. This is then reflected in a change in the relative representation of this group in the total smoker population over time. This trend is in accordance with the process in other countries, where the quicker decline in smoking prevalence was sooner reported in general population comparing to people with psychiatric comorbidity [34]. However, the England 2020 report already shows a significant decrease also in smokers with mental disorders [35]. Our herein presented data however show very high prevalence of mental disorders among smokers anyway. Although there is a well acknowledged trend to abandon smoking in the general population, the success rates in smoking cessation among those with a mental disorder remains unsatisfactory.

This study has several strengths. First of all, this is one of the largest cohorts of smokers undergoing tobacco dependence treatment with sustained biochemically verified abstinence using CO measurement in expired air. Furthermore, patients underwent repeated evaluation of depressive symptoms using BDI-II throughout the treatment.

### Limitations

As the psychiatric diagnoses were self-reported by patients, they may sometimes be incorrect or missing (i.e., withheld by the patient), resulting in an underestimation. We assume that any such underestimation would affect the data rather consistently year by year, and therefore would still allow us to track the overall relative change in prevalence over time.

We excluded all records from the years 2005, 2020 and 2021 due to consistency of our dataset: During 2005 the Centre has been established and started work, so data may be incomplete, and in 2020 and 2021 the attendance could be influenced by the COVID-19 epidemic.

## Conclusions

The results of this study suggest that a special attention should be paid to the upward trend of increasing proportion of patients with a mental disorder among smokers seeking intensive tobacco dependence treatment. These individuals should be offered a supportive environment for sharing and treating their psychiatric problems during sensitive screening. The growing proportion of smokers with a mental disorder in specialized Centre forces us to think about adjusting support tailored to these patients. They should not be stigmatised and should be entitled to require more intensive treatment for tobacco dependence alongside their psychiatric treatment. Overall, tobacco dependence treatment should be an integral part of psychiatric care.

## Data Availability

The anonymized dataset analysed during the current study is available in the OSF application repository, https://osf.io/rs5fd/?view_only=fd5c0cb924374218a1996c3757f1b02b. In case of the data request from this study, please contact the corresponding author KZ.

## Abbreviations

CI: Confidence interval
CO: Carbon monoxide
FTCD: Fagerström test for cigarette dependence
ppm: Parts per million
PRR: Prevalence rate ratio
